# Does diversity go beyond sex and gender? Gender as social category of diversity training in health profession education – a scoping review

**DOI:** 10.3205/zma001318

**Published:** 2020-03-16

**Authors:** Heidi Siller, Gloria Tauber, Margarethe Hochleitner

**Affiliations:** 1 Medical University of Innsbruck, Gender Medicine Unit, Innsbruck, Austria

**Keywords:** academic medicine, diversity, health profession, scoping review, education

## Abstract

**Background: **Sex and gender are social categories of diversity. Diversity can be perceived with an intersectional framework as it demonstrates the intersecting categories that might contribute to oppression, inequality, power and privilege. This article focused on what aspects were considered in diversity training programmes for health professions and the role of sex/gender in this context.

**Method: **This scoping review focuses on the social categories mentioned in diversity education of health professionals. Articles on diversity training for health professionals were searched for in the Web of Science database using the keywords gender, diversity, training, education and health professions. Twelve articles were finally included in this review. Thematic analysis was employed to summarise information deduced from articles.

**Findings: **Gaps in the aspects included in diversity training were identified. Findings show that culture was mostly discussed, whereas sex/gender and lesbian, gay, bisexual, transgender, queer and intersex (LGBTQI) were focused on only to a minor extent. Cultural diversity training includes self-reflection on one’s own culture, whereas a comparable tool for sex/gender and LGBTQI is missing. Additionally, other social categories of diversity, such as disability or age, are largely absent.

**Conclusion: **Diversity should be incorporated in its full breadth in health profession education and not fragmented. Additionally, other social categories such as gender might benefit from including self-reflection on these categories in addition to reflecting on the role of power and privilege in order to increase self-awareness for diversity. In this way, othering of the population might be prevented and healthcare can be improved for all.

## Zusammenfassung

**Hintergrund: **Sex und Gender sind soziale Diversitätskategorien. Diversität kann dabei mit einem intersektionalen Blick wahrgenommen werden, der aufzeigt, wie intersektionale Kategorien zu Unterdrückung, Ungleichheit, Machtunterschieden und Privilegien beitragen können. Dieser Artikel befasst sich damit, welche Aspekte in Diversitätskursen in der Ausbildung von Gesundheitspersonal berücksichtigt werden und welche Rolle Sex/Gender dabei spielt.

**Methode: **Dieser Scoping Review bezieht sich auf sozialen Kategorien, die in Diversitätskursen in Gesundheitsberufen genannt werden. Artikel zu Diversitätskursen in Gesundheitsberufen wurden in der Datenbank Web of Science anhand der Schlagwörter gender, diversity, training, education und health professions gesucht. Zwölf Artikel wurden schlussendlich in diesem Review inkludiert. Die daraus gewonnenen Informationen wurden mittels thematischer Analyse analysiert.

**Ergebnisse: **Es wurden einige Lücken bezüglich Diversitätsaspekte in Kursen festgestellt. In den Ergebnissen zeigte sich, dass der Aspekt „Kultur“ am häufigsten besprochen wurde, wohingegen Sex/Gender und lesbische, schwule, bisexuelle, transgender, queere und intersex (LGBTQI) Themen nur in einem geringen Ausmaß berücksichtigt wurden. Kulturelle Diversitätskurse inkludierten Selbstreflexion über die eigene Kultur, während vergleichbare Instrumente für Sex/Gender und LGBTQI fehlten. Zusätzlich waren weitere Diversitätskategorien wie Behinderung oder Alter weitgehend nicht vorhanden.

**Schlussfolgerung: **Diversität sollte nicht nur fragmentarisch, sondern in ihrer Gesamtheit in der Ausbildung von Gesundheitspersonal inkludiert werden. Zusätzlich könnten soziale Kategorien, wie beispielsweise Gender, von Selbstreflexion über die Bedeutung der Kategorien profitieren. Zudem sollte die Rolle von Macht und Privilegien reflektiert werden, um das Bewusstsein von Diversität zu fördern. Dabei könnte ein „othering” vermieden und Gesundheitsversorgung verbessert werden.

## Introduction

Sex and gender are emphasised as important aspects of healthcare [1], [2], [3], medical education [3], [4], [5] and medical research [3], [6], [7]. One possibility for focusing on sex and gender is to perceive these concepts as aspects of diversity, which has gained recognition in medicine over the past decades [8], often in connection with social inequality. In this sense, health professionals should reflect the diversity of the population to ensure the best possible healthcare for the population [9]. However, increasing diversity among health professionals is only one strand when providing optimal healthcare for all. An additional strand for the purpose of reacting to diversity refers to the inclusion of diversity in health profession education. In medicine, recommendations have been made on how to include various diversity aspects, for example regarding the inclusion of sex and gender [3], [10], gender diversity [11], or lesbian, gay, bisexual, transgender (LGBT) and lesbian, gay, bisexual, transgender, queer and intersex (LGBTQI) content [12], as well as diversity in general [13].

Diversity is often described in terms of ethnicity, culture, gender, sexuality, (dis)ability, socio-economic background or class and age [14]. In Europe, these social categories of diversity are also included in the EU anti-discrimination directive as a means of combatting discrimination based on sexual orientation, age, gender, religion or belief, disability or ethnicity [15]. Importantly, these social categories are connected to specific meaning in society. For example, people might be repeatedly confronted with the social meanings of these classifications (e.g. mothers and socio-cultural expectations of childcare) and might be reduced to one or more of these categories (e.g. being predominantly a mother, not a professional). Additionally, such classification might also increase the perception of difference between people based on one or two characteristics (e.g. religion, ethnicity, gender), thereby disregarding similarities between people or groups. Diversity is seen as opposed to contrasting people due to “difference” as difference obstructs the embedded context of diversity [14]. In this sense, some researchers emphasise that diversity as well as social categories of diversity should be understood in their complexity [16] and in relation to all social categories, not just to one such as ethnicity [13].

## Framework for studying diversity

Diversity is framed by an intersectional framework that provides the opportunity to elicit oppression, power, privilege and social inequality. Intersectionality was coined by Kimberlé Crenshaw [17], who demonstrated that individual social categories (e.g. ethnicity, class, gender, sexuality) have to be considered in their interwovenness in order to understand experiences of discrimination. Today, intersectionality is an important framework that encourages acknowledging the complexity of “reality” and supports an understanding of interwoven mechanisms of social inequality, but also privilege and power. Researchers focusing on intersectionality have demanded that the complexity of “reality” also be included when teaching students, e.g. by encouraging creative skills that enhance effective learning [18]. This underlines that teaching and learning should reflect the unpredictability and complexity of relations and of human (inter)actions [18]. It was also pointed out that it is necessary to engage in self-reflection to be able to include diversity in healthcare [13]. For example, self-reflection includes examining one’s own biases in terms of personal and cultural aspects as well as considering the pitfalls of stereotyping [19], but it is also necessary to become aware of one’s own racial identity [20] and privileges. Privilege is granted by birthright, e.g. ethnicity, sexual orientation, gender [21], [22], but in some instances also socio-economic status and age are considered to be privileges [21]. Thus, self-reflection is a conscious cognitive process of reflecting on behaviour, beliefs and experiences. Self-reflection is a process ranging from a pre-encounter stage to a transcendent/transcultural stage, which allows embracing and truly including diversity [20]. Even though these stages were illustrated by using race/ethnicity examples, the same effects may be detected when focusing on other categories of diversity such as gender. Therefore, students might first be skeptical and even opposed to the concept of gender before being able to critically examine the influence of gender on healthcare, interaction, everyday life and oneself. Another approach to teaching students about diversity includes self-awareness, thus students’ own narratives of diversity. Self-awareness means understanding one’s personal history, but also attitudes and values [23]. Self-awareness is employed in particular to decrease biases and stereotypes that exist towards one’s own group. Thus, it cannot be assumed that by increasing diversity in the medical workforce to reflect the given population [9], the result will be less inequality in patient care and a reduction in health disparities [24]. Teaching self-awareness and thus students’ own histories of culture, ethnicity, ableism and privileges is assumed to increase students’ awareness for their blind spots when dealing with patients or colleagues [25], [26]. Such an approach is also good when teaching cultural humility, which includes self-awareness, and self-reflection [27].

Despite several recommendations on various aspects of diversity, it is less clear how different aspects of diversity and foremost sex and gender are included in diversity education for health professionals. For this reason, a scoping review was conducted to explore the practices of including sex and gender in diversity education for health professionals. For this purpose, an intersectional framework is considered one tool for focusing on diversity. Our research was guided by the following questions:

1. what social categories of diversity are included in health profession education and, specifically, how is sex/gender as a social category of diversity included?
2. what gaps can be identified in current diversity training in health profession education?

Thus, this scoping review aims to explore the use of sex and gender in diversity training for health professionals. To discuss these questions we searched research articles for descriptions of health profession education and training offerings regarding diversity. 

## Materials and methods

The recommendations for scoping analysis as developed by Arksey and O'Malley [28] were used for this review. Scoping reviews aim to provide an overview of “the extent, range and nature of research activity” (p.21) in a given field [28]. This can also be described as a mapping of literature [29], which was intended here. To conduct a scoping review Arksey and O'Malley [28] propose several steps:

1. formulate the research question(s),
2. identify relevant studies in a given field,
3. select studies based on criteria deduced from the research question,
4. chart data as extracted from the articles,
5. collate, summarise and report results.

**Figure 1:**
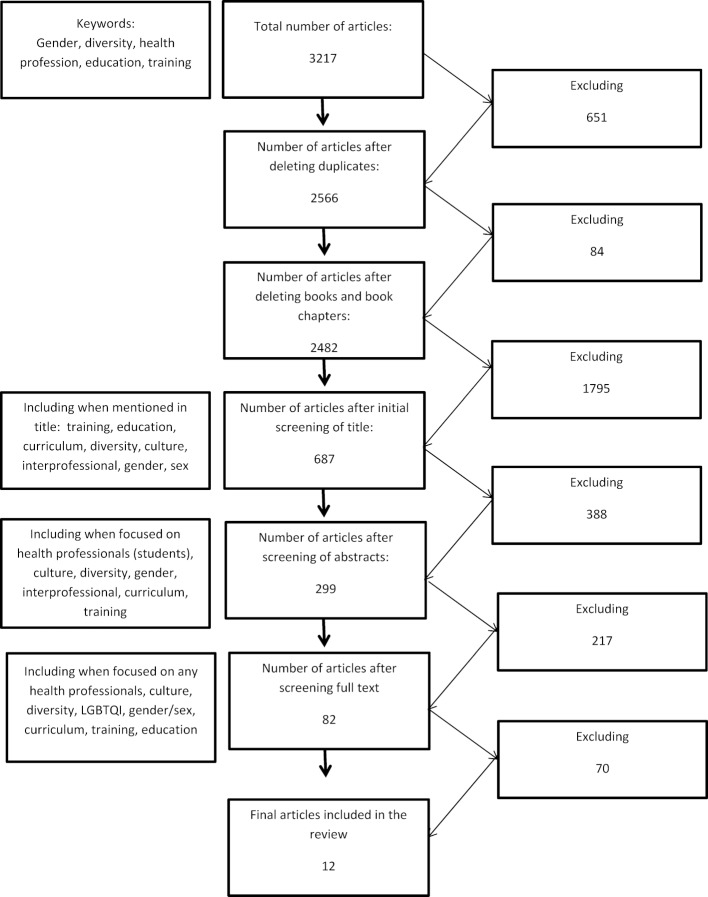
Article selection process

For the purpose of this review we searched the Web of Science database using the Web of Science Core Collection on 19 March 2018. We intended to collect a broad range of literature and to limit these search results step by step. This process is similar to that proposed by Levac, Colquhoun [29]. Thus, we initially started with a broad research question that served as the basis for our search strategy. For this purpose, we used open keywords to search the database. Keywords used in the search included: gender or diversity and health profession and education or training. These key words were used in various combinations. We limited the results by language (only English or German) and date of publication and thus used only those results published after 2000 in order to include a broad range of findings. After extracting more than 3000 articles, we narrowed these findings. In a first step we used feasibility criteria and excluded duplicates, books and book chapters, meeting abstracts or conference abstracts. From recommendations made by Levac, Colquhoun [29], we clarified our scope of research and included the clarification results in our article selection process. Thus, inclusion criteria were defined as follows:

1. the target group was health professionals or health profession students; thus, articles were included if they defined health professions according to sub-major group 22 “health professionals” of the International Classification of Occupations ISO-08 [30]. Health professionals were thus defined as medical doctors; nursing and midwifery professionals; traditional and complementary medicine professionals; paramedical practitioners; other health professionals (dentists, pharmacists, environmental and occupational health and hygiene professionals, physiotherapists, dieticians and nutritionists, audiologists and speech therapists, optometrists and ophthalmic opticians, health professionals not otherwise specified). However, not all occupations as listed here were represented in the articles. We excluded psychologists, psychotherapists and social workers as these occupations are classified as legal, social and cultural professionals (sub-major group 26), as well as other professions that form the basis of the health workforce, but are not classified in sub-major group 22 [30].We included articles that referred to health professionals/health profession students or included other professions in addition to health professionals as classified above.
2. articles had to focus on diversity or social categories of diversity in training or education of health professions (students); such a focus was given by explicitly referring to one or more social categories of diversity or naming diversity explicitly in the context of training.
3. furthermore, articles were included if authors described training content for the explicit purpose of being able to extract information about training content. These inclusion criteria were applied in several steps employed to select eligible research articles (see figure 1 (Fig. 1)).

Article selection also included reviewing all articles based on their title, thus including only those articles that dealt with education or training and mentioned these or related terms in the title. Additionally, articles had to refer to diversity, culture, gender, sex, LGBTQI or other social categories of diversity in their title. Articles were excluded if they failed to do so. A next step included consulting abstracts in order to reduce the number of articles on education or training by focusing on diversity aspects, such as gender, social class, ability, sexuality, age, culture, ethnicity as defined in the EU Anti-Discrimination Directive [15]. Finally, full texts were consulted to finalise article selection. Here, all articles that engaged in aspects other than training students or health professionals in diversity, targeted professionals other than health professionals, or did not focus on training, but on studying abroad were excluded. This also means that articles were excluded if they focused on increasing diversity in health professionals and described pipeline programmes; focused on interprofessional collaboration only; focused on training or studying diverse populations, but not health professionals; focused on professional identity, empathy or communication styles; failed to provide information on the training delivered; or provided general recommendations on how to include diversity aspects without focusing explicitly on the training content or mode of delivery, were not possible to retrieve. Ultimately, 12 articles were included in this scoping review. Steps conducted to refine results are shown in figure 1 (Fig. 1). 

Two researchers selected and summarised articles. All steps were discussed before moving to the next article selection step. If uncertain whether to exclude an article, for example if no consensus regarding inclusion/exclusion could be reached on the basis of the abstract, the full text was consulted to decide on inclusion or exclusion. 

Articles were summarised with an intersectional lens, thus focusing on intersection of social categories and its relevance in training. From the selected articles, we extracted information on the target group, the type of social category used in the training and in the article, the mode and content of training provided for the health professional students and on the question whether any outcome or improvement was found after training (see attachment 1 ). This charting of data [29] was used as a first step to categorise data retrieved from the articles and dissect the data accordingly. This procedure was based on thematic analysis [31]; we thus established several broad themes for categorising relevant information from the articles. These themes included social category of diversity, training, and evaluation (if applicable). Codes allocated to these themes were defined from information given by the articles. As a first step, we familiarised ourselves with the articles included in the review by reading and re-reading them. We subsequently selected relevant data and drafted codes accordingly. The next steps included collating the codes to the themes and reviewing themes, re-defining and sharpening the terms used for themes. We used this analysis technique to compile and discuss information on the social categories of diversity as used in these articles, training set-up and evaluations of diversity training programmes, if presented. An intersectional lens is provided as our understanding of diversity is interwoven with intersectionality. Thus, in our opinion diversity includes an understanding of interwoven, intersecting social characteristics that may affect health, access to healthcare and treatment. In this sense, intersectionality and reflexivity are important paths to acknowledging and including diversity in health systems and medical education [32]. To include an intersectional lens in illustrating the findings it is essential that the information derived from the articles be contextualised. When contextualising the information, we took into account structural, historical and social conditions. This contextualisation refers to embeddedness in the curriculum, support or resistance to diversity training; impact of diversity training on participants. 

## Results

### Social category of diversity

Diversity was connected to specific aspects of diversity and to a lesser extent was discussed in its broad range. Thus, two articles focused on lesbian, gay, bisexual and transgender (LGBT) or lesbian, gay, bisexual, transgender, queer and intersexual (LGBTQI) aspects in training or curriculum [33], [34], another two articles on sex and/or gender aspects [35], [36], two articles on diversity in general [37], [38] and six articles discussed cultural aspects [39], [40], [41], [42], [43], [44] (see attachment 1 ). A specific intersectional notion was evident in some definitions of the given social category targeted in the training programmes. In this sense, diversity was seen as interconnected categories [37] or the influence of socio-cultural interaction on gender [36]. Definitions without such a specific intersectional reference focused on the variations between groups or individuals (e.g. cultural groups) [42], set of behaviour, skills, values [38], [42] or the fluidity of concepts (e.g. gender identity) [34].

Most often, diversity was connected to culture and the acquiring of cultural competence. In this context, culture was implicitly connected to ethnicity, migration and minority groups.

Diversity appears to be included in health profession education by focusing on individual parts, but only to a small extent by focusing on the whole construct. However, it is also necessary to understand diversity in its complexity and to interconnect the individual parts of diversity as diversity is more than the sum of its parts.

#### Training and evaluation

As can be deduced from attachment 1 , a broad range of health professions were included in the training programmes (see attachment 1 ). These health professionals and health professional students participated in diversity training programmes set up as elective courses [33], [39], advanced training programmes [37] or online courses for professionals [35], [36]. However, not every article mentioned structural embedding of courses. 

In diversity training and in particular in cultural competence training, focus was not only placed on knowledge and skills, but on awareness and self-reflection. It is important that we avoid seeing culture only in “others” and that we increase cultural awareness and cultural competence by starting to dissect culture in students or staff themselves [40]. The other diversity aspects such as sex/gender [35], [36], LGBT/LGBTQI [33], [34] and at least one training course on the overall diversity approach [37] focused mainly on transfer of knowledge, increasing awareness and stimulating discussion on these subjects in science, teaching and healthcare practice. In cultural competence training programmes, self-awareness for one’s own ethnicity and culture is addressed with a view to working with a culturally diverse population, e.g. medical students caring for refugees [39], but also on a general level [38]. Writing one’s own cultural autobiography [40] was another means of fostering self-reflection on one’s own cultural values, attitudes and norms. However, changes in cultural awareness were small or moderate after training [38], [40]. These minor changes were attributed to the process of becoming self-aware [38], [40]. However, not all courses discussed in this review explicitly reported that they included such self-awareness for one’s own culture [41]. The other articles did not explicitly mention whether they included self-reflection in their training [42], [43], [44].

Other aspects of diversity, such as LGBTQI, were included in an interprofessional Health Forum [33], but were also described as lacking in a range of medical topics (e.g. mental health, chronic disease) and health professions [34]. Sex and gender aspects in health were illustrated by including these diversity aspects in diseases with known sex/gender differences (e.g. in cardiology) [35], [36] and providing this knowledge in online courses [35], [36]. Both articles used an online approach to delivering knowledge on sex and gender aspects in health and their relevance in healthcare. 

Two articles described not only gender or cultural diversity in the curriculum, but also presented diversity training programmes for health professionals concerning gender, ethnicity/race and socioeconomic status [38], as well as further diversity aspects such as sexuality, ability, religion [37]. In this way, diversity was focused on with regard to increasing awareness for diversity when providing care [37] and with regard to increasing knowledge and skills [38]. 

#### Contextualisation of diversity trainings

Including diversity and specific aspects of diversity in the training of health professionals also means having to deal with difficulties in implementing such training because of deficits in targeting the importance of diversity (aspects) in health and illness [45], and in health disparities, failure to include diversity (aspects) training in core curricula or advanced training programmes [33], [34], [36], [37], [42], [44] as well as a lack of student interest in this topic [33], [35], [38], [43], [44]. Thus, efforts to develop and implement diversity training in health professions are strongly connected to hesitation, skepticism and disaffirmation. Such attitudes are noticeable in students, but also in faculty and stakeholders. Differences in implementing courses may also stem from regional differences. Thus, some articles [38], [39], [42] mention that teaching cultural competence is a requirement, while other diversity aspects lack such support. 

## Discussion

This scoping review aims to illustrate what aspects of diversity are focused on when delivering training for health professionals, and what gaps can be identified. In total, 12 articles were included in the review. An intersectional framework is used to show that diversity is hardly included as a whole construct, but fragmented into several aspects. With Celik, Abma [37] and to some extent Ryan, Ali [42] being an exception, intersections of the social categories are often not addressed to the extent needed to illustrate the complexity of diversity. The largest share of these diversity fragments is based on culture. It can be argued that culture is not necessarily related only to ethnicity, but also encompasses all societal aspects of living together, e.g. gender roles, norms and values. However, culture is often discussed in relation to e.g. language, ethnic minority populations, cultural health beliefs [41], [44] or migration [39], [44]. However, intersections between social categories are lacking.

When teaching diversity it is also important to start with self-awareness in order to illustrate that it is not about “the others”. Another gap identified in this review relates to the neglect of other social categories of diversity, such as (dis)ability or age that fall short and are hardly discussed in this context. Even topics such as sex/gender and LGBTQI are found only to a small extent when searching for diversity in health profession education. Especially regarding sex/gender, various guidelines have been introduced in the past years that emphasise the importance of including sex/gender in research, teaching and clinical practice [1], [2], [4], [5], [6], [7]. Thus, the small extent to which sex/gender is included in diversity training programmes is surprising. In contrast, the greater extent of cultural competence in health professionals’ training programmes might be due to accreditation requirements in some countries, as mentioned by Evans and Hanes [38], Griswold, Kernan [39] and Ryan, Ali [42]. From this we see the importance of stakeholders and politics in the process of including and implementing diversity in health profession education. 

Approaches to including categories of diversity highlight that the diverse populations are perceived as the “others”, thereby othering patients and the population. This illustrates that difference is highlighted to an unnecessary extent. It should be made clear that diversity is not something to be treated in others [38], [40] but is also reflected in ourselves. Such self-reflection on and self-awareness for one’s culture were addressed in some articles [38], [40]. However, in this context especially the power and privilege of those providing healthcare need to be addressed explicitly, especially when racism, sexism, heterosexism and inequalities are discussed [32]. This was also emphasised by Griswold, Kernan [39], who pointed out that awareness for one’s privilege and power is an integral part of cultural awareness. Regardless of the mode of training, such as experiential learning or classroom teaching, self-reflection does not necessarily encompass reflecting on power and privilege, as also emphasised by Harkess and Kaddoura [45] when they state that “contact alone [does] not necessarily foster insights” (p. 220).

Additionally, self-reflection is not mentioned in articles concerning sex/gender content in health profession training and education. Therefore, it could be advantageous to also include self-reflection on and self-awareness for one’s gender role, privilege and power due to gender in order to not only cover sex/gender content in disease, but to also make it more visible when providing healthcare. Self-reflection and self-awareness are thus not restricted to cultural competence, but should be included when teaching all social characteristics of diversity [32].

This study is not without limitations. We focused on articles concerning diversity education for health professionals. However, the search strategy employed by us might have caused us to miss articles that included social categories of diversity, but did not name these diversity aspects. Furthermore, some studies might have used keywords that were not reflected in the keywords we searched for. However, we believe our main messages would remain unchanged even if other articles were included in this review. Another way to conduct this review would have been to search for various social categories of diversity to elucidate how diversity is incorporated in health profession education. However, we intended to focus on the social categories referred to when diversity education is offered to health professionals. Even though recommendations already exist on how to include diversity in health professions as well as how to include various categories of diversity (e.g. sex/gender), to our knowledge no scoping review discusses the aspects mostly considered or neglected when talking about diversity. Additionally, not all studies included an evaluation of their courses. Thus, there is still a need to study the effects of diversity courses on students, professionals and healthcare practice. Without rigorous evaluation of the curriculum, its courses, and advanced programmes we cannot ensure that students and professionals benefit from the courses in the manner they are intended to. Future research on diversity in health profession education should focus on identifying specific barriers to including diversity in health profession education. In this sense, the hidden curriculum, which transfers values, norms and other cultural subtext of a profession on an informal level and alongside the formal curriculum [46], should be considered in order to overcome such barriers. Additionally, research needs to determine what we consider diversity to be. For example, similar to research in prototypical successful academic persons envisioned as male individuals [47], diversity might be imagined as referring to culture and ethnicity, while disregarding other aspects of diversity. 

## Conclusion and implications for practice

Self-reflection and self-awareness are seen as core components of cultural competence, but have not been discussed when focusing on other social categories of diversity, such as gender. Similar to culture, gender affects every individual and is influential when it comes to health, disease and inequality [3]. Thus, gender is also not something that is present only in “others”. Self-reflection can be used as a tool to avoid othering patients, thus acknowledging and reflecting on oneself to elucidate how social categories (e.g. gender, age, ethnicity) influence oneself. This is important if we are to improve our understanding of the interaction and intersections of these categories in human (inter)action, discrimination, inequality, power and privilege and consequently to improve healthcare. This implies that diversity training should include such self-awareness in order to deconstruct health disparities, increase quality of care by socially competent health professionals, who are not only trained in treating a diverse population, but also acknowledge that they are part of the diversity. However, when focusing on diversity, it needs to be emphasised that sex and gender do not become invisible and are thus a hidden part of diversity. This also applies to all other social categories of diversity. Additionally, social categories should not be perceived as being isolated from each other. As proposed by intersectionality, inequality as well as power and privilege are influenced by intersecting social categories as well as by experiences. Such an approach also indicates that we have to reflect on the complexity and unpredictability of “reality” [18] when teaching diversity. Understanding diversity and inequality, but also providing healthcare for all, starts with reflecting on these aspects in ourselves.

## Competing interests

The authors declare that they have no competing interests. 

## Supplementary Material

Overview of studies included in scoping review
