# Supplementary material for: Does diversity go beyond sex and gender? Gender as social category of diversity training in health profession education – a scoping review
Source: GMS J Med Educ. 2020 Mar 16;37(2):Doc25. doi: 10.3205/zma001318 (PMC7171361; doi:10.3205/zma001318)
Supplement: Overview of studies included in scoping review [file JME-37-25-s-001.pdf]

Attachment 1: Overview of studies included in scoping review

No.	Author(s)	Country	Health professionals	Social category of diversity	Training	Outcome(s)/improvement from training
1.	Braun, Ramirez, Zahner, Gillis-Buck, Sheriff & Ferrone (2017)	USA	medical, dental, pharmacy, nursing, physical therapy students	LGBTQI	LGBTQI health forum including plenary sessions (skills, patient panel, introduction to terminology, documentary screening); breakout sessions (specific educational objectives); professional networking	No outcome reported
2.	Celik, Abma, Klinge & Widdershoven (2012)	The Netherlands	health professionals	Diversity (sex, gender, ethnicity, socioeconomic status, disability etc.)	Introduction; diversity and the profession, diversity and the institution, work plan (e.g. policies, guidelines)	Improved diversity awareness, knowledge, critical attitude; positive attitude towards training
3.	Evans & Hanes (2014)	USA	dental students	Culture including race/ethnicity, gender, social class	Online, interactive course on cultural competence, focusing on cultural knowledge, cultural skills and cultural attitudes. Explanation how and why health inequality evolves, thereby including diversity aspects (race/ethnicity, social class, gender)	Increase in cultural competence (overall), but only moderate increase in cultural awareness
4.	Griswold, Kernan, Servoss, Saas, Wagner & Zayas (2006)	USA	medical schools	Culture	Course consisting of storytelling sessions (refugees tell their life stories); mini clinical sessions; health education sessions; brown bag lunches	Improvements in cultural competence, communication skills
5.	Hunter & Krantz (2010)	USA	nursing students	Culture	Online or classroom format with experiential and cognitive assignments focusing on cultural awareness, cultural knowledge, cultural skills and cultural encounters	Increase was seen regarding cultural competence (overall), cultural skills, cultural knowledge, cultural desire, but not in cultural

						encounters or cultural awareness
6.	Marino R, Hawthorne L, Morgan M & Bata M (2012)	Australia	dental, medicine and physiotherapy students	Culture (transcultural skills)	Course curricula that included transcultural skills: cultural diversity concepts; transcultural and indigenous concepts of health and illness; psychosocial and cultural determinants of health; clinician-patient relationship	No outcome reported
7.	Melamed E, Wyatt LE, Padilla T & Ferry RJ (2008)	USA	college students interested in health care-related professions	Culture	Course content: introduction to cultural beliefs; medical communication, role of religion, cultural aspects of grieving; arts and music in healing; cultural perspectives from healthcare professionals; cultural competency; hospital volunteering; courses included lectures, discussions and hospital volunteering	Students appreciate the course; improvement in cultural competency based on Provider's Guide on Quality and Culture Quiz (QCQ)
8.	Muller A (2013)	South Africa	MBChB Preclinical, Occupational Therapy, MBChB Clinical, Nursing & Midwifery, Communication sciences and Disorders, Physiotherapy, Audiology	LGBT	Overview of courses covering LGBT topics; topics included in courses were: definitions of and theories on LGBT, homophobia and heterosexism; barriers to health care access; HIV and sexually transmitted infections; disorders in sex development; transitioning; sex reassignment surgery	No outcome reported
9.	Plank-Bazinet JL, Sampson A, Miller LR, Fadiran EO, Kallgren D, Agarwal RK & Cornelison TL (2016)	USA	researchers, health care providers (e.g. medical doctors, nurses, pharmacists), educators, and students in health professional schools, course is free and also open to the public	Sex and gender	Online courses; topics included are: basic science and biological basis for sex and gender difference; sex and gender in health and behaviour; influence of sex and gender on disease expression and treatment	Enhanced professional effectiveness

10.	Ryan M, Ali N & Carlton KH (2002)	USA	nursing students	Culture	An electronic networking site (web site) was implemented focusing on cultural diversity using information and case studies	Increased knowledge on impact of culture on healthcare and knowledge about healthcare in other countries. Lower agreement was given to having gained insight into one's own culture, communication with other cultural groups
11.	Seeland U, Nauman AT, Cornelis A, Ludwig S, Dunkel M, Kararigas G & Regitz-Zagrosek V (2016)	Germany	researchers, medical doctors and (medical) students	Sex and gender	eGender platform is an online learning and knowledge sharing platform that was also included in a Master Program in Public Health. Besides a basic module on Gender Medicine, seven modules discuss sex and gender aspects in various fields of medicine (e.g. cardiology, pulmonology etc.). Online learning, collaboration and face-to-face learning is provided on the platform	Satisfaction with quality and content of eGender
12.	Wagner J, Arteaga S, D'Ambrosio J, Hodge C, Ioannidou E, Pfeiffer CA & Reisine S (2008)	USA	dental medicine students	Culture	Patient-instructors with cross-cultural cues were used in mock consultations	Increase in diversity-related attitudes and behaviours; high satisfaction with the course reported
